# Prescribing memantine in general practice in England: a mixed-methods study

**DOI:** 10.3399/BJGPO.2025.0023

**Published:** 2025-12-19

**Authors:** Mary Carter, Joanne Butterworth, Chris Fox, Louise Allan

**Affiliations:** 1 University of Exeter Medical School, University of Exeter, Exeter, UK

**Keywords:** prescribing, dementia, primary health care, memantine, acetylcholinesterase, Alzheimer's disease

## Abstract

**Background:**

Acetylcholinesterase inhibitors (AChEIs) are routinely prescribed for mild-to-moderate Alzheimer’s disease (AD). National guidance advises GPs to initiate memantine for patients already taking an AChEI, as it offers small benefits for moderate-to-severe AD, with good tolerability. But this is not routinely done, potentially depriving patients of a beneficial treatment.

**Aim:**

To assess prescribing for AD in general practice, to explore factors influencing prescribing, and to identify additional education needs.

**Design & setting:**

Mixed-methods study involving GPs in England.

**Method:**

An online survey and semi-structured interviews were conducted. Survey responses were analysed in StataNow (version 18.5). Interview transcriptions were coded in NVivo (version 14) by two researchers, who agreed themes. Quantitative and qualitative analyses were integrated and mapped to the Theoretical Domains Framework (TDF) and behaviour change wheel (BCW).

**Results:**

Survey responders (*n* = 104) mostly continued rather than initiated memantine. Less than half were confident in identifying AD stages and developing care plans for moderate-to-severe AD. Overall, 46% of responders were unaware of current national guidance concerning memantine. Interviews (*n* = 23) mostly concurred with survey findings. Direction from local formularies conflicts with current national guidance. Mapping to TDF and BCW identified barriers to, facilitators, and interventions for changing practice.

**Conclusion:**

Limited time, patchy support, and Quality and Outcomes Framework downgrading contribute to a perception that dementia is not prioritised in general practice. Local systems for diagnosis and treatment reinforce GPs’ feelings of inadequacy. GPs assess the impact of AD on patients and families but may not map assessments to a disease stage for memantine initiation. Interventions to change practice should boost knowledge and confidence; local pathways should clearly reflect national guidance.

## How this fits in

Current National Institute for Health and Care Excellence (NICE) guidance allows GPs to prescribe memantine for moderate-to-severe Alzheimer’s disease (AD) in patients already taking an acetylcholinesterase inhibitor (AChEI) without referral to secondary care. However, prescribing rates suggest this is not routine, potentially depriving patients of beneficial treatment. This mixed-methods study with GPs in England explored factors influencing AD prescribing and education needs. While GPs assess AD’s impact on patients, they may not align assessments with the stage for memantine initiation. With competing priorities and uneven support for dementia care, interventions should focus on boosting knowledge and confidence in a range of contexts. Local pathways should align with national guidance.

## Introduction

Dementia affects an estimated one in 14 people aged >65 years and one in six people aged >80 years, with an estimated total of >670 000 patients with dementia in the UK^1^ and >55 million worldwide.^2^ The UK figure is projected to increase to 1.4 million by 2040.^3^

AD is the most common cause of dementia, and comprises 70% of individuals with a dementia diagnosis in the UK.^3^ AD is a progressive disease with a range of cognitive and behavioural symptoms.^4^ Approximately 70% of people with an AD diagnosis require admission to a care home.^5^ Global assessment measures, such as the Clinical Dementia Rating (CDR) scale,^6^ identify stages of AD severity and guide treatment decisions and the choice of pharmacological therapies, which are approved for different stages of the disease. In trials reporting CDR global dementia scores, a score of 1 denotes ‘mild’, 2 indicates ‘moderate’, while 3 suggests ‘severe’ AD. In 2020, the Alzheimer’s Society estimated that moderate and severe AD accounted for 28% and 14% of all diagnoses, respectively.^7^

Initial diagnosis of dementia in the UK is provided by secondary care specialists, including psychiatrists, geriatricians, and neurologists working in memory clinics or services. GPs may continue initiated treatments under a shared care protocol. These treatments include AChEIs (for example, donepezil, galantamine, and rivastigmine), which are prescribed individually to manage mild-to-moderate AD. AChEIs prevent acetylcholinesterase from breaking down acetylcholine, increasing its concentration in the brain. This enhances nerve cell communication, which may temporarily alleviate or stabilise symptoms.^8^

Memantine, a N-methyl-D-aspartate receptor antagonist, which prevents excess glutamate from causing damage to the nerve cells in the brain,^8^ is safe and may be effective in treating dementia of all severities.^5^ Although memantine has little effect on mild AD, it demonstrates a small improvement for cognitive and functional symptoms in patients with moderate-to-severe AD,^9,10^ but not on neuropsychiatric symptoms.^11^ Adding memantine to treatment with AChEIs may slow down cognitive deterioration and have a beneficial effect on the severity of behaviour and mood problems in patients with moderate-to-severe AD.^5^ Although usually well-tolerated, side effects may include sleepiness, dizziness, headaches, constipation, and shortness of breath. Memantine, when used in conjunction with donepezil (an AChEI), may help extend the lives of patients with AD by as much as 5 years.^12^

According to the most recent (2018) NICE guidance,^13^ prescription for memantine may be initiated by a GP for a patient with moderate-to-severe AD, if the patient is already taking an AChEI,^13^ without referral back to secondary care. However, rates of prescription suggest that GPs are not yet routinely prescribing memantine, thereby potentially depriving patients of the benefits of a disease-modifying medication.

This study aimed to establish the situation with respect to prescribing for AD in general practice, to explore factors influencing GPs’ practice, and to establish the need for additional education to support a change in practice.

### Research questions

This study addressed the following research questions:

- To what extent are GPs in England following current NICE guidance^13^ concerning prescribing memantine for patients with moderate-to-severe AD?
- What are the barriers to and facilitators for following the guidance for this group of patients?
- What educational or training needs do GPs have with respect to prescribing memantine for patients with moderate-to-severe AD?

## Method

This study adopted a mixed-methods^14^ approach, comprising the following components.

### Online survey

An online survey was developed using Qualtrics XM and piloted with four clinicians. For estimating the proportion of GPs who were initiating memantine, a minimum of 100 participating GPs were required to achieve a 10% margin of error at a 95% confidence interval.

The survey was made available between February and September 2024 via integrated care boards, local medical committees, Health Innovation Networks, Applied Research Collaborations, professional groups, for example, the Society for Academic Primary Care and WiseGP,^15^ and social media from health-related X (formerly Twitter) and LinkedIn accounts.

Before accessing survey questions, responders viewed an online participant information sheet and completed a checkbox to consent to participate. The survey comprised 15 questions to gather data on responder demographics, their general practice, and their experience of prescribing and care for patients with AD. The interview topic guide is included in Supplementary X.

#### Analysis

Survey responses were exported to StataNow (version 18.5) for cleaning and analysis. Data from GPs who provided consent and completed the survey questions were included in the analysis.

### Qualitative interviews

The survey was used to invite the responder to participate in a subsequent online or telephone interview. A target sample matrix was developed to guide recruitment of a varied sample. We emailed responders to set up interviews.

#### Analysis

Interview transcripts were imported into QSR NVivo (version 14) for coding. The interviewer (non-clinical researcher) transcribed all interview recordings, thoroughly reviewed each transcript, and developed a codebook^16^ of preliminary labels (codes) for the data. A second researcher (academic GP) examined and coded a sample of the transcripts. The codes were discussed by the two researchers and jointly structured into themes, which were assessed and adjusted to ensure they accurately reflected the data. The researchers incorporated reflexivity^17^ into this process, aiming to reduce the impact of assumptions and biases.

#### Integration of results

Results of quantitative and qualitative analysis were integrated to identify factors influencing GPs' care and prescribing for moderate-to-severe patients with AD and mapped to the Theoretical Domains Framework (TDF),^18^ behaviour change wheel (BCW), and intervention functions.^19^ Interventions grounded in robust theories of human behaviour are more likely to lead to changes in practice,^20^ and the TDF and BCW provide a clear framework for understanding current practices, barriers to, and facilitators for following NICE guidance, with which to inform future education, training, and changes to practice in AD patient care.

This research reflects Leech and Onwuegbuzie’s guidelines for conducting and reporting mixed research.^21^

## Results

A total of 115 GPs consented to participate in the survey, but 11 did not complete it. Data from 104 GPs were available for analysis.

Tables 1 and 2 summarise details of interviewees and survey responders, respectively. Supplementary Table S1 contains all survey results.

**Table 1.** Characteristics of GPs and practices (interview participants)

Individual characteristic	*n*
Has initiated memantine	
Yes	5
No	18
Sex	
Male	13
Female	10
Years working as a GP	
0–4	1
5–9	5
10–14	7
15–20	10
Special interest in dementia	
Yes	7
No	16
Mode of interview^a^	
Online	17
Telephone	6
**Practice characteristic**	
Number of GPs in practice^b^	
1–5	3
6–10	8
>10	9
Deprivation^b,c^	
≤5	12
>5	8
Region	
East of England	3
London	2
North East	2
North West	6
South East	3
South West	3
West Midlands	2
Yorkshire & Humberside	2

^a^Interviews conducted April–September 2024; average length of interview = 29 minutes and 45 seconds (range 17.43–54.54). ^b^ *n* = 3 not known. ^c^IMD deprivation decile^35^ of general practice (lower number indicates greater deprivation). IMD = Index of Multiple Deprivation.

**Table 2.** Characteristics of GPs and practices (*N* = 104 responders)

**Characteristic**	***n***	**%**
**Sex**		
Male	50	48.1
Female	51	49.0
Prefer not to say	3	2.9
**Years since qualifying as a GP**		
0–4	12	11.5
5–9	24	23.1
10–14	23	22.1
15–19	13	12.5
20–24	12	11.5
≥25	20	19.2
**Number of sessions^a^ worked per week**		
1–2	9	8.7
3–4	23	22.1
5–7	53	51.0
≥8	16	15.4
**Working as a locum**		
No	78	75.0
Yes	26	25.0
**Special interest in dementia**		
No	73	70.2
Yes	31	29.8
**Practice location**		
East of England	10	9.6
East Midlands	4	3.8
London	20	19.2
North East England	2	1.9
North West England	21	20.2
South East England	9	8.7
South West England	26	25.0
West Midlands	4	3.8
Yorkshire and Humberside	7	6.7
**Number of GPs in practice**		
1–5	20	19.2
6–10	49	47.1
11–15	25	24.0
16–20	2	1.9
21–25	4	3.8
26–30	3	2.9

^a^One session = 4 hours and 10 minutes.^36^

Results from the survey and interviews are presented in themes relating to care and prescribing for patients with AD in general practice under the following category headings: 1) the wider healthcare context; 2) practice or organisational factors; and 3) individual factors.

### Wider healthcare context

#### Existing division of responsibilities

Overall, 46% (46.2%, *n* = 48) of survey responders and a similar proportion of interviewees were unaware of the 2018 NICE guidance regarding memantine.^13^ Most interviewees gave precedence to local formulary recommendations, while 46.2% (*n* = 48) of survey responders reported that they ‘regularly’ referred patients with moderate-to-severe AD to specialists. Most interview responders reported that, according to their local formulary classifications, memantine is assigned ‘amber’ status. This classification indicates that only specialists (geriatricians and psychiatrists) should initiate memantine. GPs are recommended to continue this treatment under a shared care agreement.

Reflecting the established division of responsibilities between general practice and specialist care providers, only 14.4% (*n* = 15) of survey responders regularly initiated memantine, while 63.5% (*n* = 66) regularly continued a memantine prescription.

Many GPs did not separate their views about adopting additional responsibilities for treatment (for example, for initiating memantine for their patients with moderate-to-severe AD) from existing processes for diagnosing dementia in their locality. Some GPs accepted the logic of the existing division of responsibilities:

*'I don't see most GPs where I work wanting to initiate this, if there’s already a service available … not wanting to initiate it before they’d been formally assessed, because you need to get that diagnosis first and memantine may hinder that process. I'm not quite sure how it would fit into our current work in practice.'* (Male [M], 5–9 years since registering as a GP, Yorkshire and Humberside [YH], Index of Multiple Deprivation [IMD] decile 2)

However, other GPs questioned existing practices and believed that adjustments to responsibilities in general practice could benefit their patients:

*'If we're not confident with a drug … if we haven't done it often, we're a bit fearful of it … it also goes hand in hand with this sort of mystique around the diagnosis itself. So those who would benefit from memantine … are … medium to severe. And often with the right sort of story … it would be completely reasonable to make the diagnosis without having to put them through stuff* [such as being referred back to specialist care]*.'* (Female [F], 15–20 years, South East England [SE], IMD decile 5)

Some, usually more experienced, GPs had acted against local practice and initiated memantine themselves:

*'I really don't know what our formulary says, but I've been using memantine for quite a long time really.'* (M, 15–20 years, South West England [SW], IMD decile 8)

Many GPs used screening tools only as part of local procedures for accessing specialist services and believed that time pressure, even with a double appointment, precluded in-depth assessments needed to determine dementia severity. Several were not complimentary about the standard assessment tools available to GPs (for example, the 6-Item Cognitive Impairment Test,^22^ General Practitioner’s Assessment of Cognition,^23^ and the Mini-Mental State Examination^24^) and mentioned the challenge of using standard screening tools to assess patients with differing pre-morbid intellectual capacities. Some GPs were unable to use certain tools because licences had not been renewed in their area.

The interval between referral of a patient from general practice and an appointment at a memory clinic mostly ranged from 3–6 months. Referral criteria varied and some GPs were critical about delays:

*'Nightmare situation that we have … a service that takes months to get an appointment … then eventually after about six months after the referral we get a diagnosis. I don't see why GPs can't do it.'* (F, 15–20 years, North West England [NW], IMD decile 5)

#### Availability of support

GPs acknowledged that they were dependent on a range of community-based services to support and supplement their care for people with dementia. The local frailty team, community dementia nurses, and voluntary organisations (for example, Admiral Nurses) were often mentioned. There were gaps in some locations, whereas in others these services had taken the lead. Navigating complicated systems that were subject to changes in staff and remit could be difficult:

*'In our area we've got community dementia nurses who will, when we've made a diagnosis … go out and see the patient and family and talk about the diagnosis and driving and living wills and support groups. So that’s very useful. I think if we haven't got that, then that would make our life very much more difficult.'* (M, 15–20 years, SW, IMD decile 8)

Most survey responders had access to a specialist for advice about patients with AD (69.2%, *n* = 72). Interviewees reported a range of experiences in this respect: from responsiveness and collaborative working, to frustration with the unavailability of specialists or those in a sufficiently senior role. Several GPs identified NHS advice and guidance,^25^ integrated into clinical systems (for example, EMIS and SystmOne), as a useful source of specialist support. However, availability varied by location.

Two GPs with an extended role (GPwERs) in dementia provided insights from their dual role in general practice and in specialist memory clinics. One mentioned changes to her scope of practice following postgraduate training:

*'I probably do most of the prescribing in my own GP surgery for* [dementia and frailty] *... I would previously have referred back to the old age psychiatry team or linked back in with the specialist services before changing the medication. I wouldn't have felt confident, even though it’s in the NICE guidelines, I just didn't have the experience of doing it.'* (F, 5–9 years, SE, IMD decile 6)

The other recognised the reasons for poor-quality dementia assessments provided by GPs to the memory clinic:

*'That’s largely two things, one, GPs don't have enough time to do a proper assessment. But secondly, they very often don't think to ask the sort of functional questions that add weight to the memory issue.'* (F, 15–20 years, SE, IMD decile 5)

GPs noted increased use of new Additional Roles Reimbursement Scheme^26^ staff, often shared across primary care networks, who supported people with AD and their families while maintaining up-to-date knowledge of local resources:

*'In our practice we're really fortunate we've got a really good system of social prescribing. We've got a wellbeing coordinator with an interest in elderly and mental health issues. So I would be keen to … get them* [patients with AD] *hooked into one of those services, get that up and running and monitoring … '* (M, 15–20 years, YH, IMD decile 3)

### Practice or organisational factors

#### Access and continuity of care

Almost all interviewees expressed a strong belief in the importance of continuity of care for this patient group, although many felt unable to offer it in their general practice. They asserted that prior knowledge of individuals and their families was essential to accurately assessing the impact of cognitive decline:

*'When new stuff crops up, physical health, mental health, social stuff, it’s absolutely critical … to know the background, to know where you started from, to make an assessment of what’s going on.'* (M, 10–14 years, North East England [NE], IMD decile 1)

GPs from smaller practices were more often able to provide continuity of care for their patients. Several GPs acknowledged that continuity of care had been compromised by the prevalence of part-time working in general practice. In some cases, continuity was provided by a consistent group of people, rather than an individual GP:

*'I don't think that continuity needs to just come from one person … as part of a team around the patient. So for example, a frailty nurse that really knows that patient or care home nurse, in addition to the GP. I think that’s really important.'* (F, 5–9 years, SW, IMD decile 9)

Some GPs spoke about asking patients to attend for two consultations, so that a thorough assessment could be made, before referring to a memory clinic. However, owing to widespread pressure on access to general practice, GPs reported that it was often difficult to book a further appointment with the same GP.

Some practices in areas of greater deprivation had prioritised continuity:

*'I think we're all very keen that our patients trust us and given the area, with sort of high levels of deprivation and high distrust of professionals and service, it’s important that* [dementia] *review comes from someone that they know and trust … I think that’s quite reassuring for the patient … but also the carer.'* (M, 10–14 years, NW, IMD decile 1)

#### Competing priorities

Some GPs spoke about the pressure to respond to a range of competing priorities in their general practice, and the tendency to engage with conditions and issues that were incentivised by associated funding via the Quality and Outcomes Framework (QOF). Several GPs noted the recent downgrading of the QOF dementia indicator and associated payments from 39 to 19 possible points,^27^ and suggested that new initiatives or changes in dementia care would require additional funding. Many believed that dementia was not afforded the high profile it deserves:

*'It feels as if dementia is not getting as much of the attention as it needs … it is getting more prevalent … So it then means that we need to give more care, be more aware and, because the treatment and the management of dementia is mostly proactive, anticipatory, then you have to have been thinking about those things earlier on.'* (F, 0–4 years, NE, IMD decile 1)

Most GPs had some responsibilities for patients with AD living in care homes and emphasised the importance of good relationships with care home staff. This was sometimes facilitated by a partnership between an experienced member of care home staff and an identified ‘link GP’. One GP expressed frustration with the local specialist multidisciplinary care team, which made decisions about moderate-to-severe dementia care without first-hand knowledge of the patients:

*‘We have to fill in a form and then another nurse … does a 2-hour assessment. And then they take it to this big meeting, where they have a big old chat and none of them actually met the patient … sometimes the expertise of people who are on the ground seeing them all the time and knowing the family and knowing the nuances is lost.’* (M, 10–14 years, NE, IMD decile 1)

### Individual factors

#### Confidence, knowledge, and skills gaps

Many GPs believed that they had become somewhat deskilled by the process of referring patients to secondary care for memory assessment, but opportunities for training were not consistently available. Most GPs were open to the idea of training in dementia care, and they identified a wide range of topics as the focus of any potential training, including local referral conventions, legal and capacity issues, and improving diagnosis skills. A minority of survey responders reported being ‘very confident’ or ‘confident’ about assessing the stage of AD for a patient (39.4%, *n* = 41) or about developing a care plan for a patient with moderate-to-severe AD (42.3%, *n* = 44). Less than half ‘regularly’ (41.3%, *n* = 43) or ‘occasionally’ (36.5%, *n* = 38) conducted annual reviews with patients with moderate-to-severe AD.

Although most GPs recognised gaps in their knowledge in respect of dementia, they often mentioned a lack of time in which to receive training. They had differing preferences about the format of training and upskilling. Some favoured a ‘top-down’ approach, whereby all GPs in a location could receive the same training:

*'We have patient educational time-out sessions ... one a month … local CCG* [clinical commissioning group] *group-led ones … that would be the perfect opportunity for them to educate us. Educate every single GP in the local area.'* (F, 15–20 years, NW, IMD decile 5)

Others suggested an online forum to which they could bring queries about dementia care to discuss with colleagues in their locality:

*'We have something called Clinicians Connect … a Microsoft Teams chat channel. All day long that’s been having things posted on it about … "I don't know what to do about this. Does anyone else have any thoughts?" And it’s not just the GPs. We've got our pharmacists … ANPs* [advanced nurse practitioners] *… nursing staff … physios. Everyone’s on it … I mean the amount of learning that comes out of it!'* (F, 15–20 years, SE, IMD decile 4)

Some GPs mentioned online sources of information they had independently accessed, such as Red Whale and eLearning for Healthcare, and through their membership of the Royal College of General Practitioners.

The two GPwER interviewees strongly valued the particular perspective their role afforded. Several other interviewees believed that they would benefit from gaining first-hand experience of a specialist dementia setting:

*'It might be quite nice to sit into one of the … memory clinic assessments and just seeing our colleagues at work and you know how they assess it and what sort of things they go through, that might be interesting.'* (M, 15–20 years, East of England, IMD decile 5)

#### Patient-centred care

Many GPs were dissatisfied with the primacy afforded to specialist diagnosis of dementia, which they believed undervalued their holistic understanding of patients and ability to address a wide range of aspects impacted by AD:

*'I don't think this is about diagnosis per se … therefore it’s not about a single condition, whether it’s dementia or hypertension or arthritis or whatever. It’s about how we make sense with the person of their daily life.'* (F, 15–20 years, NW, IMD decile 1)

GPs contrasted these skills with services offered by specialist teams, who had less familiarity with individual patients and their families:

*'I think the main* [thing] *is the centrality of holism, generally and sometimes the difficulties I have with specialists are that people are in their silos, so the psychiatrist is focused on just psychiatry and the geriatricians … and actually it sometimes feels like me and my nurse colleague are the only ones that are taking the whole patient into account, and I think that’s a really valuable perspective.'* (M, 10–14 years, NE, IMD decile 1)

#### Prescribing versus non-pharmacological care

Several GPs were concerned about interactions between dementia medications (including memantine) and other medications taken by their patients. Others felt concerns about interactions were overemphasised, leading patients to stop effective, ‘first-line’ medications (for example, amitriptyline) for dementia drugs with limited benefits:

*'But you know the sort of risk is phenomenally low, and I wonder sometimes if we're not giving people the best treatment because of it*.*'* (M, 10–14 years, West Midlands [WM], IMD decile 8)

GPs had often gained experience of memantine in a care home setting and expressed a range of views about its tolerability and effectiveness:

*'I use it* [memantine] *in care homes … when I feel that people are moving from … mild to moderate to severe … in certain cases, it can be helpful.'* (F, 5–9 years, SE, IMD decile 6)

*'I know there’s evidence for them of some kind of modest benefit. But I've seen … quite a few patients in the care home who have had symptoms which have stopped when we've taken them off them … the nurses in the care home really don't like memantine at all.'* (M, 10–14 years, NE, IMD decile 1)

Several GPs observed that patients in care homes are often prescribed too many medications and identified deprescribing as one of their key responsibilities.

Several responders were critical about what they perceived as an emphasis on pharmacological responses to the problems presented by AD:

*'It frustrates me sometimes that so much resource goes into medication when actually I think the things that would change and improve people’s lives and ultimately their deaths, because often a lot of people* [with] *dementia are or would be … on our gold standard framework … towards the end of their life, are not medicines and those are the things that really do help people.'* (M, 5–9 years, WM, IMD decile 9)

Table 3 presents themes on AD care and prescribing in general practice, including facilitators for and barriers to memantine use, mapped to TDF domains, BCW components, and potential functions to guide interventions for modifying GP practice.

**Table 3.** Themes mapped to TDF domains, BCW components, and potential function categories

Theme	Facilitators for prescribing memantine in general practice	Barriers to prescribing memantine in general practice	TDF domain(s)	BCW component(s)	Potential function categories
Existing division of responsibilities	Change to formulary advice to match NICE NG97	Formulary continues to direct GP to specialist for treatment initiation	Environmental context and resources	Physical opportunity	Environmental restructuring
Availability of support	GP has good access to specialist and other support Multidisciplinary team working is effective	Specialist and other support unavailable Multidisciplinary team working is absent or not effective	Environmental context and resources Beliefs about capabilities	Physical opportunity Reflective motivation	Environmental restructuring Education
Access and continuity of care	Patients with AD can easily book appointments with the same GP	Practice cannot offer continuity of care for patients with AD	Social or professional role and identity Beliefs about consequences Environmental context and resources	Reflective motivation Physical opportunity	Education Persuasion Enablement Modelling Environmental restructuring
Competing priorities	Practice identifies dementia as a priority	Practice follows QOF designation of dementia	Environmental context and resources Reinforcement	Physical opportunity Automatic motivation	Environmental restructuring Training Incentivisation
Confidence, knowledge, and skills gaps	Information about NICE (NG97) guidance Training in prescribing memantine and care for moderate-to-severe AD Confidence in evidence supporting memantine	Lack of information about NICE (NG97) guidance No training or not available in preferred format	Knowledge Skills Beliefs about capabilities Beliefs about consequences	Psychological capability Reflective motivation	Education Training Persuasion Enablement Modelling
Patient-centred care	Belief that GP has the skills and is appropriate for prescribing for AD	Belief in current division of responsibilities between GP and specialists	Knowledge Skills Social or professional role and identity Beliefs about capabilities	Psychological capability Reflective motivation	Education Training Persuasion Enablement Modelling
Prescribing versus non-pharmacological care	Low concerns about memantine in terms of effectiveness, tolerability, and interactions Knowledge of memantine	Concerns about memantine Lack of knowledge about memantine	Beliefs about capabilities Beliefs about consequences Knowledge Optimism	Reflective motivation Psychological capability	Education Persuasion Enablement Modelling

AD = Alzheimer's disease. BCW = behaviour change wheel. NICE = National Institute for Health and Care Excellence. QOF = Quality and Outcomes Framework. TDF = Theoretical Domains Framework.

## Discussion

### Summary

This study found varied responses to the division of responsibilities in dementia care and prescribing. Factors within the wider healthcare context, and at practice or organisation and individual levels influenced GPs' attitudes and behaviour towards the balance of responsibility for this patient group. The limited time, use of basic tools (compared with those employed by specialists) for dementia assessments, and downgrading in the QOF reflect a perception that dementia is not a priority in general practice. Furthermore, current direction from local formularies concerning responsibilities for managing medications are typically at variance with guidance from national bodies such as the NICE. As such, local systems for referral, diagnosis, and treatment initiation reinforce some GPs’ feelings of inadequacy in respect of their patients with moderate-to-severe AD. They may not feel capable of offering a full range of treatment options, particularly if support from within and outside the practice is not available. GPs customarily assess the growing impact of AD on patients and families, but may not align assessments with the stage at which memantine initiation is appropriate. These factors perpetuate the existing division of responsibilities.

### Strengths and limitations

The exploratory survey results were confirmed and explained by in-depth investigation of issues of interest and importance relating to the care of patients with dementia in the one-to-one interviews. Survey participants were recruited from all areas of England and included a reasonable spread of sex, years of general practice experience, and size of general practice. Recruiting interview participants via the survey was effective, leading to interviews with GPs of varied experience, perspectives, and patient populations. Participants could choose a telephone or online interview at their convenience, ensuring flexibility and removing barriers to participation.

GP networks, websites, and social media accounts were used to promote and distribute the survey, so it was not possible to calculate a true response rate and generalisability of survey findings is unclear. Purposively recruiting interviewees based on survey responses may have provided further insights, but this was not feasible.

To our knowledge, this is the first known mixed-methods study to explore GPs’ perspectives on prescribing memantine for moderate-to-severe AD in general practice. The qualitative component provides rich data regarding the range of factors influencing GPs’ current practice. Mapping these factors to the TDF and BCW has suggested potential ways in which the status quo can be challenged and modified by future interventions.

### Comparison with existing literature

To our knowledge, this is the first study with the specific purpose of understanding factors that influence GPs’ behaviour and attitudes in respect of prescribing in general practice for patients with moderate-to-severe AD. GPs work within multi-layered healthcare contexts, and prescribing is influenced by factors that operate in the wider healthcare environment,^28^ the general practice,^29^ and within the individual GP.^30^ Our findings suggest that changes in the balance between specialist care and general practice require addressing the sources of various influences, particularly guidance from local formularies.

Mapping to the theoretical constructs of the TDF and BCW has been used previously to identify and describe factors that influence GP prescribing behaviour, and to inform the development of interventions aimed at modifying prescribing practice.^31,32^ In our study, three TDF domains featured prominently: 'Environmental context and resources', 'Knowledge', and 'Beliefs about capabilities’. These domains echo findings from previous mappings of determinants of prescribing for older people in primary care.^33,34^

### Implications for practice

Further mapping of the TDF domains identified in our study suggests that interventions to modify GPs’ prescribing practice should incorporate diverse educational approaches, including guidance on memantine from credible specialists, to boost both knowledge and confidence in respect of prescribing for moderate-to-severe AD. Given the varying priorities among general practices and uneven access to internal and external support for dementia care, it is important to consider the wider healthcare context in which GPs make prescribing decisions for this patient group. Local pathways should reflect national guidance and need to be clearly articulated.

## Data

The dataset relied on in this article is available from Open Research Exeter.
